# Identification of metabolism genes related to hepatocarcinogenesis and progression in type 2 diabetes mellitus via co-expression networks analysis

**DOI:** 10.1186/s41065-021-00177-x

**Published:** 2021-04-17

**Authors:** Yiming Bi, Bei Yin, Guanjie Fan

**Affiliations:** 1 School of Second Clinical Medicine, Guangzhou University of Chinese Medicine, Guangzhou, China; 2 Department of Endocrinology, Guangdong Provincial Hospital of Chinese Medicine, the Second Affiliated Hospital of Guangzhou University of Chinese Medicine, Guangzhou, China

**Keywords:** Type 2 diabetes mellitus, Hepatocellular carcinoma, Metabolism genes, WGCNA

## Abstract

**Background:**

Type 2 Diabetes Mellitus (T2DM) is an independent risk factor of hepatocellular carcinoma (HCC). However, the related genes and modules to hepatocarcinogenesis and progression in T2DM remain unclear.

**Methods:**

The microarray data from Gene Expression Omnibus (GEO) were analyzed to screen differentially expressed genes (DEGs) of T2DM and HCC dataset. Then, weighted gene co-expression network analysis (WGCNA) was performed on these DEGs to detect the modules and genes, respectively. Common genes in modules with clinical interests of T2DM and HCC were obtained and annotated via GOSemSim package and Metascape. Genes related to late-stage HCC and high glycated haemoglobin (HbA1c) were also identified. These genes were validated by UALCAN analysis and univariate cox regression based on The Cancer Genome Atlas (TCGA). Finally, another two independent datasets were applied to confirm the results of our study.

**Results:**

A total of 1288 and 1559 DEGs of T2DM and HCC were screened, respectively. Kyoto Encyclopedia of Genes and Genomes (KEGG) enrichment revealed several shared pathways in two diseases, such as pathways in cancer and metabolism. A total of 37 common genes correlated with T2DM and HCC were then identified with WGCNA. Furthermore, 12 genes from modules associated with late-stage HCC and high HbA1c were regarded as hub genes. Among these genes, 8 genes associated with tumor invasion and metastasis were validated by UALCAN analysis. Moreover, downregulations of *ACAT1*, *SLC2A2*, *PCK1* and *ABAT* were significantly associated with poorer prognosis in HCC patients with elevated HbA1c. Additionally, the expressions of *PCK1* and *ABAT* were raised in HepG2 cells pre-treated with metformin and phenformin.

**Conclusions:**

The present study confirmed several metabolic genes related to hyperglycemia and malignant tumor, which may provide not only new insights into the pathogenesis of hepatocarcinogenesis and progression in T2DM, but also novel therapeutic targets for T2DM patients with HCC in the future.

**Supplementary Information:**

The online version contains supplementary material available at 10.1186/s41065-021-00177-x.

## Background

Type 2 diabetes mellitus (T2DM) is one of the most common chronic diseases worldwide. According to International Diabetes Federation (IDF), diabetes affected an estimated 463 million people worldwide in 2019, accounting for 9.3 percent of adults aged 20 to 79, with a predicted increase to 10.9 percent by 2045 [1]. Besides eliciting metabolic disorders and vascular damages, T2DM is a major predisposing factor of cancers, predominantly in gastrointestinal malignancies, including hepatocellular carcinoma (HCC) [2]. Accordingly, approximately 7 percent of new cases of HCC can be attributed to diabetes, and the incidence of HCC in patents with T2DM is about twofold higher than that in normal individuals [3, 4]. The risk of HCC development is also positively correlated with T2DM duration, which significantly increases to 7.52 times in individuals with a 10-year-duration of diabetes [5].

Furthermore, there is also strong evidence indicating the relationships between the rise of the incidence of T2DM and the risk of deaths from HCC [6]. A meta-analysis showed that the presence of diabetes decreased overall survival rate among patients with HCC; and the mortality for HCC was 2.5-fold higher in T2DM patients [7]. A number of studies also reported that treatment with glucose-lowering medications like metformin could improve the survival rate of patients with HCC [8]. These shreds of evidence revealed that T2DM had turned into an independent risk factor for HCC development.

The underlying mechanisms involved in T2DM and HCC are complicated, including hyperglycemia, insulin resistance, and inflammatory [9]. Endogenous insulin acting on liver activates a number of signaling pathways such as insulin-like growth factor (IGF) signaling pathway, phosphatidylinositol 3 kinase (PI3K) pathways and mitogen-activated protein kinase (MAPK) pathways that contribute to hepatic cell proliferation and tumor progression [10]. Further, persistently high blood glucose and circulating insulin levels accelerate the secretion of inflammatory factors and the accumulation of metabolites such as free fatty acids (FFA) and glycation end products (AGEs). As a result, hepatic stellate cells are activated and liver fibrosis is promoted [11]. Chronic hyperglycemia also increases the frequency of *KRAS* and *MYC* variants, possibly because of nucleotide imbalance [12]. Although the metabolism disorders of T2DM have been reported to play crucial roles in stimulating liver cancer growth, the specific pathological mechanisms and key genes of these processes remain vague.

Weighted gene co-expression network analysis (WGCNA) is a novel biological method widely utilized in the high-throughput sequencing data analysis. It focuses on the intramodular connectivity and gene significance, which alleviates the multiple testing problem inherent in microarray [13]. Based on the co-expression networks, the hub genes for relating modules to one another and to external clinic traits are identified. This method has been successfully applied to identify shared pathogenesis of 2 diseases. For instance, Zhu et al. used WGCNA to reveal ten hub genes involved in the development of Alzheimer's disease and T2DM [14]. Bi et al. also employed WGCNA to demonstrate a novel biomarker for distinguishing alcohol-associated HCC from non-alcohol-associated HCC [15]. Thus, to fully understand T2DM and HCC, we utilized this method to search for genes acting on metabolism disorders in T2DM and development in HCC.

## Materials and methods

### Data preparation

Gene expression datasets GSE38642, GSE44035 and GSE25724 were downloaded from GEO, which contains 70 normal samples and 16 T2DM samples. Meanwhile, GSE101685 was collected, including 8 normal samples and 24 HCC samples. All data in different samples were normalized by quantity prior to performing gene differential analysis with limma R package [16]. Differentially expressed genes (DEGs) with p-value < 0.05 were screened. DEGs with |logfold change (logFC)|> 0.2 in T2DM and |logFC|> 1 in HCC were further investigated in our study. In addition, two independent datasets, GSE50397 and GSE69850, were downloaded to validate the results of the present study. GSE50397 provided 89 samples with different levels of blood glucose (HbA1c), while GSE69850 provided an evaluation of changes in gene expression associated with the treatment of human HepG2 cells with 34 different chemical compounds, including metformin and phenformin.

### Gene ontology (GO) and KEGG analysis of DEGs

In order to explain the biological functions and further interactions, both DEGs of T2DM and HCC were annotated by GO analysis and KEGG pathways analysis. The *p* value < 0.05 was set to be statistically significant, with the top 10 visualized in R.

### Weighted gene co-expression networks (WGCNA) and module analysis

The DEGs of T2DM and HCC utilized WGCNA R package to construct co-expression networks with corresponding clinic traits, respectively [13]. Data were checked to identify the outliers in the samples. All samples from T2DM dataset were well clustered, while one offending sample was removed in HCC dataset. The soft-thresholding power identified by pickSoftThreshold function was applied to the automatic network construction. The result was clustered by topological overlap matrix analysis, containing module assignments labeled in colors and module eigengenes (MEs). In addition, the correlations between MEs and clinic traits were calculated via Pearson's correlation test. The modules with a |ME|> 0.3 and a *p*-value < 0.05 were considered momentous in the interactions with clinical features [17].

### Correlation analysis between clinically significant modules in T2DM and HCC

Furthermore, genes relevant to T2DM and HCC within each module were annotated via GO biological process (BP) enrichment. Different sets of GO terms in modules of T2DM and HCC were collected. Since the functions of genes can be enriched by GO terms, their biological function similarities can also be assessed by aggregating similarities of GO term sets. Therefore, the biological correlations between T2DM and HCC modules were determined by the calculation of GO semantic similarities via DOSE R package [18]. The result with a correlation score ≥ 0.5 was defined as significant, indicating that there was a certain correlation in biological function between the two modules.

### Common genes identification and analysis in T2DM and HCC

The DEGs within related modules were analyzed with Venn tool (https://www.bioinformatics.org/. psb.ugent.be /webtools/Venn/) to define the common upregulated and downregulated genes in T2DM and HCC. Common genes involved in the pathogenesis of T2DM and HCC were interpreted by Metascape (last updated on 2021–02-01) [19], a website tool integrating ontology sources such as GO biological process, KEGG pathways, Reatome gene sets, Canonical Pathways, DisGeNET and PaGenBase.

### Prognostic genes identification and validation

In addition, the correlations between cancer progression and HbA1c abnormality were also explored. Genes in corresponding modules were screened from common genes in T2DM and HCC. With a gene significance (GS) > 0.2 and module membership (MM) > 0.8 [17], hub genes were selected and further validated by UALCAN analysis based on TCGA database (http://ualcan.path.uab.edu/analysis.html) [20]. Univariate Cox regression was also applied to detect high risk factors in HCC. Moreover, the protein samples of prognostic genes were also validated via the Human Protein Atlas (http://www.proteinatlas.org).

### Hub genes confirmation via external independent data

To confirm the correlations with prognostic genes and glucose control, genes were analyzed in an independent dataset GSE50397. According to the HbA1c value, T2DM samples were divided into 2 sub-units (HbA1c > 6.5% and HbA1c ≤ 6.5%), and one-way ANOVA was made to compare the genes expressions among normal, and the 2 sub-units of T2DM. Finally, the correlations between hyperglycemia and prognostic genes were also validated through HepG2 samples prepared with hypoglycemic agents.

## Results

### Identification and functional enrichment of DEGs

**Fig. 1:**
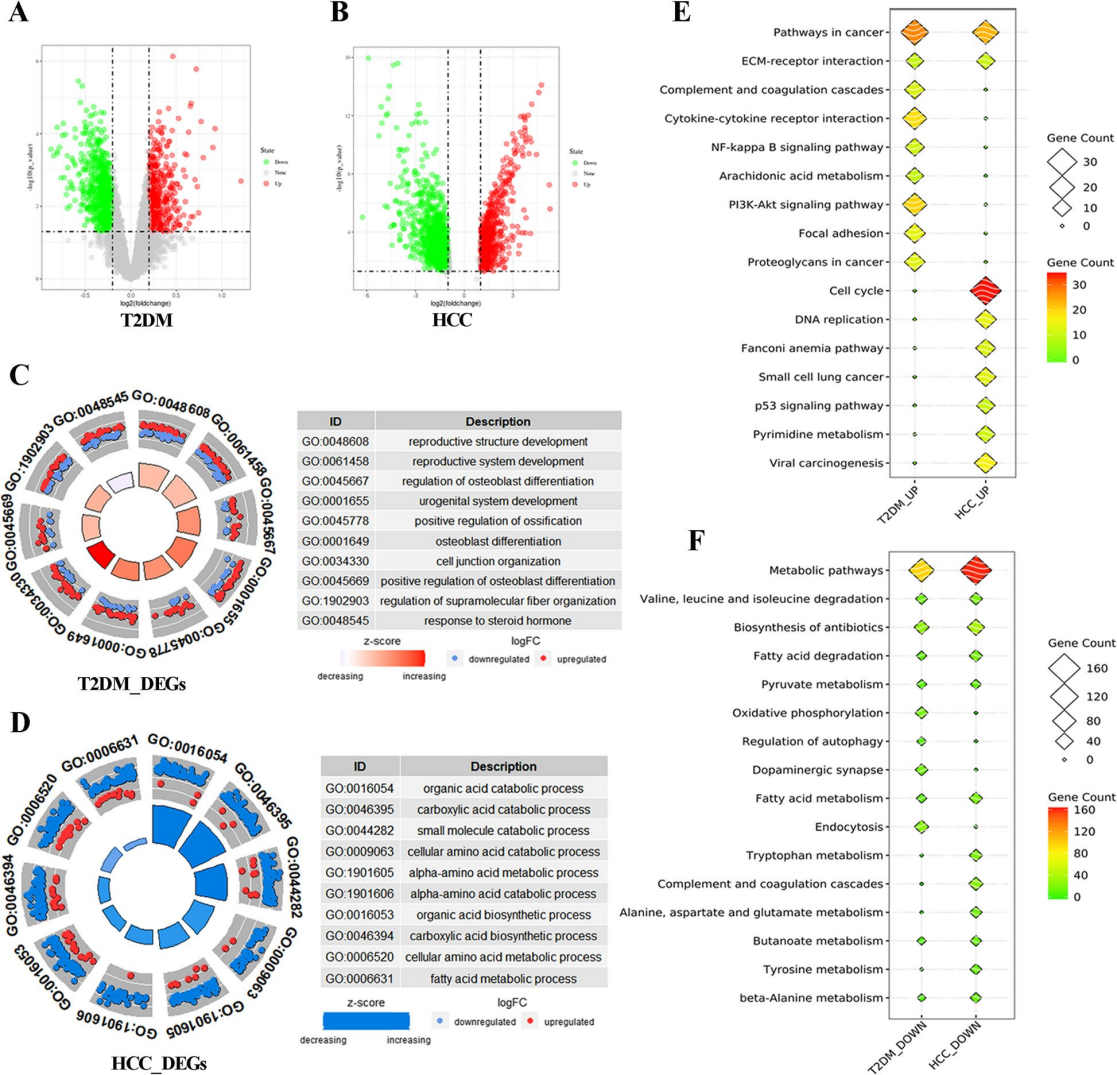
**a** and **b** The volcano plots of T2DM and HCC. **c** and **d** The GO terms in T2DM and HCC. **e** and **f** The KEGG analysis of upregulated genes and downregulated genes in T2DM and HCC

A total of 1288 DEGs were screened between T2DM and normal controls, whereas 1559 DEGs were obtained between HCC and the normal samples. Of these DEGs, 440 genes were upregulated and 848 were downregulated in T2DM, while 618 were upregulated and 941 were downregulated in HCC (Fig. 1a and b). As indicated by GO analysis, DEGs in T2DM were mainly enriched in reproductive structure development and cell junction organization (Fig. 1c). On the other hand, metabolic processes involving oranic acid, carboxylic acid and small molecules were observed in HCC (Fig. 1d). KEGG analysis exhibited several shared pathways concerning T2DM and HCC. The upregulated DEGs in T2DM and HCC were enriched in pathways in cancer and extracellular matrix (ECM)-recptor interaction, whereas the downregulated DEGs were enriched in metabolic pathways and amino acids degradation (Fig. 1e and f). In brief, both GO and KEGG analyses strongly demonstrated the correlations between T2DM and HCC, since the expression of genes involved in cellular metabolic was significantly reduced, and the expression of genes associated with cell development was substantially increased. The detailed information of functional enrichment of DEGs is shown in Supplementary Table S1 to S6.

### Co-expression networks construction and modules correlation between T2DM and HCC

**Fig. 2:**
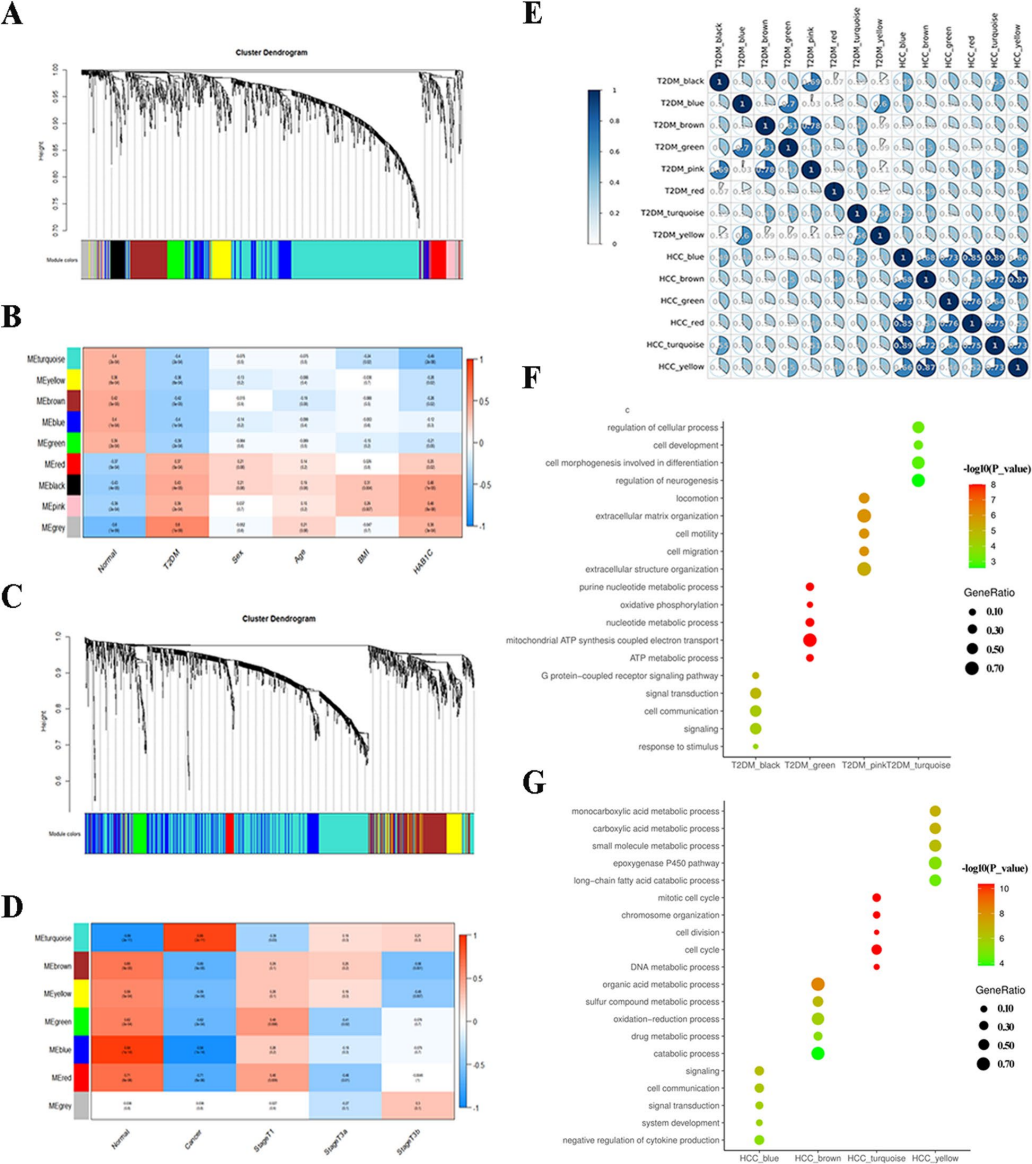
**a** and **b** Hierarchical clustering dendrogram of T2DM and heatmap plot of correlation between modules and clinical traits of T2DM. **c** and **d** Hierarchical clustering dendrogram of HCC and heatmap plot of correlation between modules and clinical traits of HCC. **e** The correlation analysis between each module in T2DM and HCC. **f** and **g** The GO analysis of genes in modules related to T2DM and HCC

WGCNA was utilized to screen gene clusters with similar biological functions and construct the correlations between gene clusters and specific clinic traits. A soft power of β = 7 was set and eventually a total of 9 gene clusters, also known as modules, were identified in T2DM. Additionally, a total of 7 modules in HCC were obtained with a soft power of β = 8. The gray module represented the non-clustering genes and was excluded. Genes in yellow, blue, turquoise, brown and green modules were negatively correlated with T2DM, while those in black, pink and red modules were positively correlated (Fig. 2a and b). The result of HCC demonstrated that all modules except for the turquoise one were negatively correlated with HCC (Fig. 2c and d). The top 10 GO BP terms of each module in 2 diseases were collected (Supplementary Table S7-S8). These GO terms that summarized the biological functions in each module were analyzed by DOSE package to determine the correlations between T2DM and HCC. As a result, the biological functions of black and pink modules in T2DM focused on cell communication and cell motility, which were similar to those in turquoise module in HCC. Likewise, genes within turquoise and green modules of T2DM were enriched in cell development and cellular metabolism, exhibiting the similar actions to those in blue, brown and yellow modules in HCC (Fig. 2e to g). These modules were considered as significant modules relevant to T2DM and HCC, and were managed with further study. Then, 4 upregulated genes and 33 downregulated genes were obtained through the overlap of several significant modules (Table 1). These 37 genes were annotated via Metascape database, and the result exerted their ability to regulate cellular metabolic processes and liver development (Supplementary Table S9). The associations between genes and diseases were also revealed by DisGeNET (Table 2). The results exhibited that these 37 core genes were highly associated with hypoglycemia, diabetes mellitus, liver carcinoma and steatohepatitis, which were consistent with our study.

**Table 1:** Thirty-seven genes correlated with HCC development in T2DM

Gene	Term	T2DM		HCC	
		LogFC	*P*_value	LogFC	*P*_value
CXCL5	C-X-C Motif Chemokine Ligand 5	0.40	7.57E-03	1.65	1.95E-02
GPC3	Glypican 3	0.39	6.27E-03	5.22	5.70E-08
NETO2	Neuropilin And Tolloid Like 2	0.22	8.83E-03	1.39	1.14E-03
IGF2BP2	Insulin Like Growth Factor 2 MRNA Binding Protein 2	0.22	4.10E-02	1.43	1.98E-02
ACAT1	Acetyl-CoA Acetyltransferase 1	-0.33	7.41E-04	-1.47	4.35E-04
CRYL1	Crystallin Lambda 1	-0.29	1.18E-03	-1.20	4.34E-03
SLC2A2	Solute Carrier Family 2 Member 2	-0.65	2.54E-03	-1.88	1.13E-02
PCK1	Phosphoenolpyruvate Carboxykinase 1	-0.33	4.89E-02	-3.96	3.27E-04
ABAT	4-Aminobutyrate Aminotransferase	-0.50	2.75E-03	-1.91	1.15E-02
ACADSB	Acyl-CoA Dehydrogenase Short/Branched Chain	-0.29	7.88E-04	-1.76	2.39E-03
ST3GAL6	ST3 Beta-Galactoside Alpha-2,3-Sialyltransferase 6	-0.27	2.55E-02	-2.15	1.29E-06
EPHX2	Epoxide Hydrolase 2	-0.32	1.07E-02	-1.96	4.06E-04
KCNMA1	Potassium Calcium-Activated Channel Subfamily M Alpha 1	-0.34	2.10E-02	-1.93	3.94E-07
SORL1	Sortilin Related Receptor 1	-0.42	1.01E-02	-2.36	1.36E-07
ACYP2	Acylphosphatase 2	-0.25	1.51E-03	-1.09	3.55E-03
QDPR	Quinoid Dihydropteridine Reductase	-0.26	4.17E-02	-1.12	1.43E-02
TSPAN7	Tetraspanin 7	-0.52	3.92E-03	-1.22	5.81E-03
MAN1C1	Mannosidase Alpha Class 1C member 1	-0.29	9.13E-03	-1.74	2.16E-05
SC5D	Sterol-C5-Desaturase	-0.39	9.94E-04	-1.61	3.96E-03
IRS2	Insulin Receptor Substrate 2	-0.21	1.99E-02	-1.45	4.61E-03
RBL2	RB Transcriptional Corepressor Like 2	-0.27	1.10E-02	-1.09	8.00E-03
F8	Coagulation factor VIII	-0.20	2.28E-02	-1.23	6.47E-04
PRKAR2B	Protein Kinase CAMP-Dependent Type II Regulatory Subunit Beta	-0.30	9.63E-03	-1.56	1.25E-03
ETFDH	Electron Transfer Flavoprotein Dehydrogenase	-0.27	1.80E-02	-1.61	3.04E-05
LIMCH1	LIM And Calponin Homology Domains 1	-0.29	4.39E-02	-1.21	1.78E-02
HADH	3-Hydroxyacyl-CoA Dehydrogenase	-0.33	8.97E-03	-1.19	4.69E-04
ACSL1	Acyl-CoA Synthetase Long Chain Family Member 1	-0.43	1.36E-03	-1.63	4.18E-03
PTS	6-Pyruvoyltetrahydropterin Synthase	-0.21	4.82E-02	-1.20	1.83E-04
PHLDA2	Pleckstrin Homology Like Domain Family A Member 2	0.29	8.18E-03	1.47	2.22E-02
GAS2	Growth Arrest Specific 2	-0.28	4.53E-02	-1.09	1.28E-02
TGFBR3	Transforming Growth Factor Beta Receptor 3	-0.45	2.95E-03	-1.25	1.96E-03
PEMT	Phosphatidylethanolamine N-Methyltransferase	-0.21	4.74E-02	-1.43	4.27E-04
PTPN3	Protein Tyrosine Phosphatase Non-Receptor Type 3	-0.33	1.76E-03	-1.07	2.35E-03
PPP1R1A	Protein Phosphatase 1 Regulatory Inhibitor Subunit 1A	-0.74	5.26E-05	-1.59	2.55E-02
RNF130	Ring Finger Protein 130	-0.22	1.90E-03	-1.24	2.20E-02
RCBTB2	RCC1 And BTB Domain Containing Protein 2	-0.30	9.65E-03	-1.26	3.23E-05
OAT	Ornithine Aminotransferase	-0.49	8.27E-04	-2.50	1.86E-03

**Table 2:** The relationship between 37 genes and diseases via DisGeNET analysis

Term	Category	*P*_value	Enrichment
Muscle hypotonia	DisGeNET	3.98E-06	8.3
Hypoglycemia	DisGeNET	2.51E-05	24
Diabetes mellitus, experimental	DisGeNET	3.16E-05	22
Liver carcinoma	DisGeNET	5.01E-05	12
Steatohepatitis	DisGeNET	5.01E-05	41
Seizures	DisGeNET	6.31E-05	6.9
Global developmental delay	DisGeNET	6.31E-05	6.8
Cognitive delay	DisGeNET	6.31E-05	6.8
Mental and motor retardation	DisGeNET	6.31E-05	6.8
Obesity	DisGeNET	1.00E-04	11

### Hub genes identification and validation relevant to HCC progression and HbA1c abnormality

**Fig. 3:**
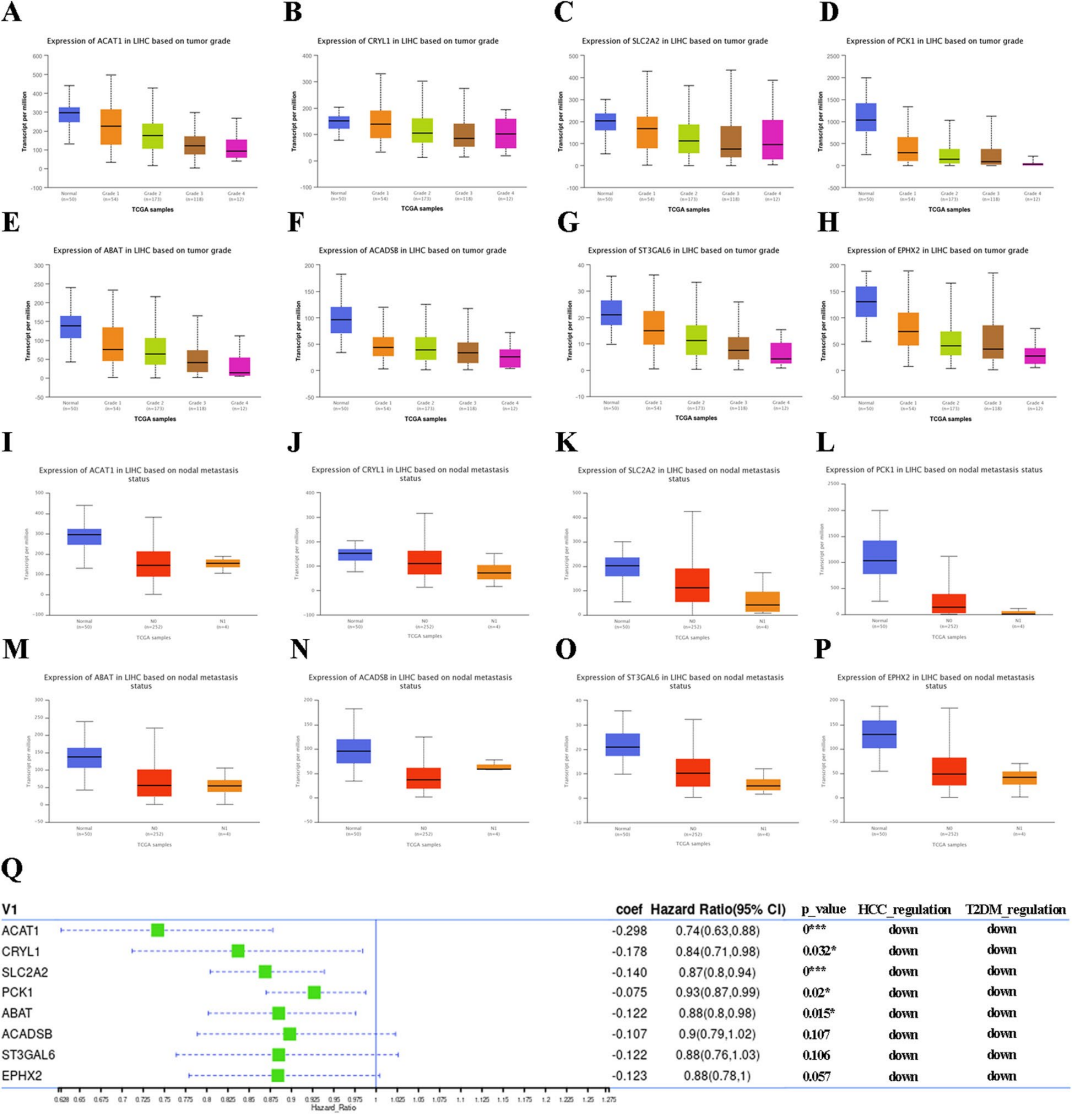
**a** to **h** The expressions of *ACAT1*, *CRYL1*, *SLC2A2*, *PCK*, *ABAT*, *ACADSB*, *ST3GAL6* and *EPHX2* based on tumor grade. **i** to **p** The expressions of *ACAT1*, *CRYL1*, *SLC2A2*, *PCK1*, *ABAT*, *ACADSB*, *ST3GAL6* and *EPHX2* based on metastasis status. **q** The correlation between genes expression and HCC survival

Notably, genes in the brown and yellow modules negatively associated with StageT3b of HCC and genes in the turquoise cluster negatively correlated with HbA1c of T2DM were also analyzed. A total of 24 genes were identified, of which 12 were selected as hub genes related to tumor invasion and HbA1c variability. Eight genes, including *ACAT1*, *CRYL1*, *SLC2A2*, *PCK1*, *ABAT*, *ACADSB*, *ST3GAL6* and *EPHX2* were validated to be negatively correlated with tumor stage and nodal metastasis status based on TCGA database (Fig. 3a to p). Moreover, univariate cox regression analysis was performed to calculate the hazard ratio (HR) between genes and survival status of HCC. Genes with HR < 1 were defined as protective factors, and the results demonstrated that the increased expressions of five genes, *ACAT1*, *CRYL1*, *SLC2A2*, *PCK1* and *ABAT*, might be beneficial to improve the prognosis of HCC (Fig. 3q).

### Prognostic genes confirmation

**Fig. 4:**
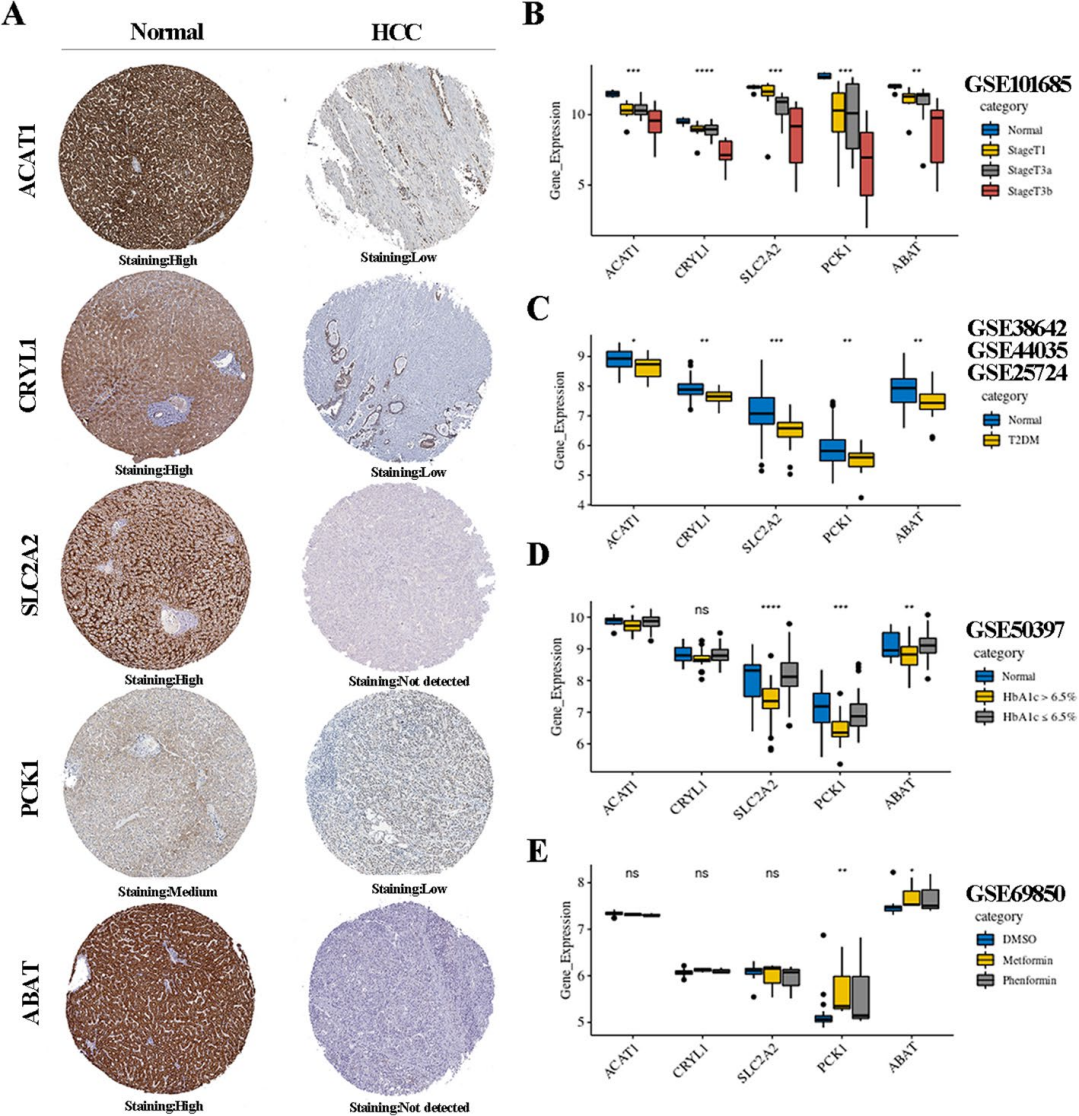
**a** The expression level of prognostic genes in HCC and normal samples. **b** The expression level of prognostic genes in HCC dataset. **c** The expression level of prognostic genes in T2DM datasets. **d** The expression level of prognostic genes in another T2DM dataset GSE50397. **e** The expression level of prognostic genes in another HCC dataset GSE69850

The protein expressions of five genes were validated between HCC tissue and normal liver tissue via Human Protein Atlas. The expressions of *ACAT1*, *CRYL1*, *SLC2A2*, *PCK1* and *ABAT* appeared to be lower in HCC issue than in normal tissue (Fig. 4a and b). Moreover, the expressions of *ACAT1*, *SLC2A2*, *PCK1* and *ABAT* in T2DM samples with HbA1c > 6.5% were lower than those in normal samples and the samples with HbA1c ≤ 6.5% (Fig. 4c and d). Besides, *PCK1* and *ABAT* were significantly improved in HepG2 samples prepared with metformin and phenformin in GSE69850, suggesting that glycemic control in T2DM might be beneficial for improving survival outcomes in HCC by increasing the expressions of protective factors (Fig. 4e).

## Discussions

Liver is a major metabolic hub, and aberrant metabolism resulted from T2DM leads to a spectrum of liver dysfunctions such as fatty liver, cirrhosis and even hepatocellular carcinoma. Besides, patients with clinical T2DM characteristics generally influence future HCC and liver-related mortality, particularly those with poorly controlled diabetes [21]. Due to the poor prognosis and unsatisfactory life expectancy in patients with HCC, it is of great clinical significance to clarify the molecular mechanisms of HCC development in T2DM patients.

In this study, key modules and genes involved in T2DM development and HCC growth were observed by bioinformatics method. Through the analysis, it was found that genes were enriched in cell communications and ECM organization in several significant modules of two diseases; additionally, the staining results showed the increasing deposition of collagen type IV and α-smooth muscle actin (α-SMA) in liver of T2DM patients, indicating that hepatic ECM remodeling with overnutrition played a crucial role in liver malignancy [22]. On the other hand, we found that the activity of catabolic progress was decreased in both T2DM and HCC. The pyruvate metabolism and fatty acid metabolism involved in the progress of glycolysis or gluconeogenesis were concurrently enriched in two diseases; not only that, the degradation of amino acid was reduced. These changes may facilitate the biosynthesis and proliferation of cancer cells, which are known as metabolic reprogramming in cancer. Metabolic reprogramming is a hallmark of malignancy, and precedes liver cancer with oncogene mutation [23]. The results of our study suggested that glucose and glutamine metabolism in T2DM was the leading cause of HCC-associated metabolic reprogramming. With further study, 37 genes closely associated with carcinogensis in T2DM were revealed, and the interactions among them were concentrated on carbohydrate metabolism as well. These findings are consistent with several published studies [24, 25], and provide a promising direction for the investigation of the metabolism-related molecules relevant to the development of HCC and progression of T2DM.

Currently, it has been demonstrated that pool glycemic control (assessed by HbA1c) exacerbates HCC. Accumulating evidence indicated that participants with HbA1c ≥ 6.5% had an increased risk of cancer mortality and postoperative tumor recurrence [26, 27]. The signature in this research was based on metabolic genes, which were not only significantly associated with the growth of HCC, but also exerted prediction of the progression and prognosis of HCC in T2DM patients with higher HbA1c. In present study, we found eight genes including *ACAT1*, *CRYL1*, *SLC2A2*, *PCK1*, *ABAT*, *ACADSB*, *ST3GAL6* and *EPHX2* were influenced by HbA1c and related to tumor metastasis. Moreover, the majority of these genes performed on the steps of glutaminolysis, ketogenesis, and molecular transport. Further validation via another database revealed that 4 hub genes, *ACAT1*, *SLC2A2*, *PCK1* and *ABAT*, were correlated with tumor survival of HCC in T2DM with HbA1c ≥ 6.5%, suggesting the prognostic prediction function of these 4 genes and even new therapeutic targets in HCC.

Among these genes, acetyl-CoA acetyltransferase 1, encoded by *ACAT1*, is an enzyme that regulates ketone metabolism based on different energy status, and downregulation of *ACAT1* is an important feature in the pathophysiology of type 2 diabetes [28]. The decreased *ACTA1* in late-stage HCC may be caused by metabolic changes, and a number of results showed that over-expression of *ACAT1* inhibited the proliferation and migration of tumor cells [29]. However, another study indicated that inhibition of *ATAC1* retarded tumor formation in mice combination with sorafenib [30]. The complicated energetics in tumor may be the cause of these contradictory results and it seems that the anti-tumor effect of *ACAT1* needs to be further investigated. As a product of *SLC2A2*, glucose transporter 2 is regarded as a glucose sensor due to its low affinity for glucose. The suppression of *SLC2AS* leads to impaired insulin secretion. Studies have indicated that the expression of *SLC2A2* is replaced by SLC2A1 in HepG2 cells, resulting in enhanced nutrient uptake and cells proliferation [31]. Phosphoenolpyruvate carboxykinase 1, a post transcriptional protein of *PCK1*, catalyzes the first rate-limiting reaction of gluconeogenesis in liver, which is closely linked to the tricarboxylic acid cycle (TCA) flux. Numerous studies have proven the anti-tumor effects of *PCK1*. It has been reported that the viability of HepG2 cells is raised through knocking down *PCK1*, while increasing the expression of *PCK1* activates adenosine 5′-monophosphate-activated protein kinase (AMPK) and c-Jun pathways, blocks gluconeogenesis, and promotes TCA cataplerosis, leading to cell cycle arrest and tumor cells death [32, 33]. Moreover, the elevated *PCK1* inhibits the migration in two HCC cell lines and the growth of solid tumor in nude mouse xenograft models [34]. *ABAT* is a gene encoding 4-aminobutyrate aminotransferase. The absence of *ABAT* in HCC leads to the accumulation of γ-amino butyric acid (GABA), which promotes the growth of HCC in vitro and in vivo [35]. Recently, *ABAT* is also essential for mitochondrial nucleoside metabolism, and its dysfunctions enhance cellular nucleoside imbalance and in turn accelerate DNA mutations [36, 37]. Furthermore, mitochondrial nucleoside replication induced by *ABAT* deficiency also promotes one-carbon metabolism remodeling, which may contribute to cisplatin resistance and cell migration of HCC [38]. Finally, we detected the expressions of prognostic genes in HepG2 cells pre-treated with metformin and phenformin. Interestingly, the expressions of *PCK1* and *ABAT* were increased, suggesting that *PCK1* and *ABAT* were not only the prognostic biomarkers, but also the therapeutic targets of metformin and phenformin in T2DM and HCC. The result was consistent with several studies suggesting that metformin remarkably suppressed the growth of *PCK1*-knockout tumor cells and inhibited tumor growth in an orthotropic HCC mouse model [33].

## Conclusions

In summary, T2DM-associated susceptibility modules and genes for HCC were revealed through co-expression network analysis. Four key metabolism genes *ACAT1*, *SLC2A2*, *PCK1* and *ABAT* were identified from T2DM patients with poorer glycemic control. The dysfunctions of these genes may affect the anabolism and catabolism of substances such as glucose, fatty acids and amino acids, leading to changes in energy sources in cells, and further contributing to the proliferation and migration of HCC. These findings may provide a rational explanation for the higher morbidity and poorer prognosis of HCC in T2DM patients. Additionally, our study has also demonstrated that biguanides may regulate *PCK1* and *ABAT* to achieve therapeutic ends within T2DM and HCC. However, the potential of these genes as effective targets for energy regulation based anti-HCC therapies needs to be verified in more experiments and clinical practice in the future.

## Supplementary Information

**Additional file 1**:**Table S2**. The top 100 GO analysis of DEGs in T2DM. **Table S2**. The top 100 GO analysis of DEGs in HCC. **Table S3**. The top 20 KEGG analysis of upregulated DEGs inT2DM. **Table S4**. The top 20 KEGG analysis of downregulated DEGs inT2DM. **Table S5**. The top 16 KEGG analysis of upregulated DEGs in HCC. **Table S6**. The top 30 KEGG analysis of downregulated DEGs in HCC. **Table S7**. The top 10 GO analysis of each module in T2DM. **Table S8**. The top 10 GO analysis of each module in HCC. **Table S9**. The top 100 functional analysis of 37 genes via Metascape.

## Data Availability

The data of this study are available via corresponding databases mentioned within the article.
