# Supplementary material for: Identification of metabolism genes related to hepatocarcinogenesis and progression in type 2 diabetes mellitus via co-expression networks analysis
Source: Hereditas. 2021 Apr 17;158:14. doi: 10.1186/s41065-021-00177-x (PMC8053303; doi:10.1186/s41065-021-00177-x)
Supplement: Supplementary file 1 — Additional file 1:Table S2. The top 100 GO analysis of DEGs in T2DM. Table S2. The top 100 GO analysis of DEGs in HCC. Table S3. The top 20 KEGG analysis of upregulated DEGs inT2DM. Table S4. The top 20 KEGG analysis of downregulated DEGs inT2DM. Table S5. The top 16 KEGG analysis of upregulated DEGs in HCC. Table S6. The top 30 KEGG analysis of downregulated DEGs in HCC. Table S7. The top 10 GO analysis of each module in T2DM. Table S8. The top 10 GO analysis of each module in HCC. Table S9. The top 100 functional analysis of 37 genes via Metascape. [file 41065_2021_177_MOESM1_ESM.doc]

Table S1 The top 100 GO analysis of DEGs in T2DM

| ID | Term | Category | P_value |
| --- | --- | --- | --- |
| GO:0048608 | reproductive structure development | BP | 3.69E-05 |
| GO:0061458 | reproductive system development | BP | 3.69E-05 |
| GO:0045667 | regulation of osteoblast differentiation | BP | 6.32E-05 |
| GO:0001655 | urogenital system development | BP | 6.39E-05 |
| GO:0045778 | positive regulation of ossification | BP | 1.34E-04 |
| GO:0001649 | osteoblast differentiation | BP | 1.34E-04 |
| GO:0034330 | cell junction organization | BP | 1.73E-04 |
| GO:0045669 | positive regulation of osteoblast differentiation | BP | 1.97E-04 |
| GO:1902903 | regulation of supramolecular fiber organization | BP | 1.97E-04 |
| GO:0048545 | response to steroid hormone | BP | 3.15E-04 |
| GO:0007548 | sex differentiation | BP | 3.84E-04 |
| GO:0007015 | actin filament organization | BP | 4.16E-04 |
| GO:0048667 | cell morphogenesis involved in neuron differentiation | BP | 4.64E-04 |
| GO:0010810 | regulation of cell-substrate adhesion | BP | 4.73E-04 |
| GO:0032970 | regulation of actin filament-based process | BP | 4.90E-04 |
| GO:0045137 | development of primary sexual characteristics | BP | 4.90E-04 |
| GO:0034329 | cell junction assembly | BP | 5.08E-04 |
| GO:0051493 | regulation of cytoskeleton organization | BP | 5.08E-04 |
| GO:0050673 | epithelial cell proliferation | BP | 5.08E-04 |
| GO:0050678 | regulation of epithelial cell proliferation | BP | 5.08E-04 |
| GO:1901136 | carbohydrate derivative catabolic process | BP | 5.08E-04 |
| GO:0072001 | renal system development | BP | 5.08E-04 |
| GO:0044282 | small molecule catabolic process | BP | 5.08E-04 |
| GO:0030278 | regulation of ossification | BP | 6.59E-04 |
| GO:0030073 | insulin secretion | BP | 7.03E-04 |
| GO:0032956 | regulation of actin cytoskeleton organization | BP | 7.20E-04 |
| GO:0001503 | ossification | BP | 7.20E-04 |
| GO:0001822 | kidney development | BP | 7.42E-04 |
| GO:0007409 | axonogenesis | BP | 9.50E-04 |
| GO:1901888 | regulation of cell junction assembly | BP | 9.94E-04 |
| GO:0032535 | regulation of cellular component size | BP | 1.06E-03 |
| GO:0008406 | gonad development | BP | 1.06E-03 |
| GO:0031589 | cell-substrate adhesion | BP | 1.66E-03 |
| GO:0030316 | osteoclast differentiation | BP | 1.66E-03 |
| GO:0006027 | glycosaminoglycan catabolic process | BP | 1.77E-03 |
| GO:0046879 | hormone secretion | BP | 1.77E-03 |
| GO:0090066 | regulation of anatomical structure size | BP | 1.78E-03 |
| GO:0042326 | negative regulation of phosphorylation | BP | 1.83E-03 |
| GO:0010721 | negative regulation of cell development | BP | 2.22E-03 |
| GO:0030099 | myeloid cell differentiation | BP | 2.22E-03 |
| GO:0006026 | aminoglycan catabolic process | BP | 2.22E-03 |
| GO:0110053 | regulation of actin filament organization | BP | 2.22E-03 |
| GO:0051017 | actin filament bundle assembly | BP | 2.43E-03 |
| GO:0061564 | axon development | BP | 2.43E-03 |
| GO:0001952 | regulation of cell-matrix adhesion | BP | 2.43E-03 |
| GO:0030072 | peptide hormone secretion | BP | 2.43E-03 |
| GO:0061572 | actin filament bundle organization | BP | 2.53E-03 |
| GO:0001933 | negative regulation of protein phosphorylation | BP | 2.57E-03 |
| GO:0009914 | hormone transport | BP | 2.74E-03 |
| GO:0050796 | regulation of insulin secretion | BP | 2.84E-03 |
| GO:0044272 | sulfur compound biosynthetic process | BP | 2.84E-03 |
| GO:0051384 | response to glucocorticoid | BP | 2.84E-03 |
| GO:0002573 | myeloid leukocyte differentiation | BP | 2.84E-03 |
| GO:0072073 | kidney epithelium development | BP | 3.10E-03 |
| GO:0010975 | regulation of neuron projection development | BP | 3.10E-03 |
| GO:0016054 | organic acid catabolic process | BP | 4.08E-03 |
| GO:0046395 | carboxylic acid catabolic process | BP | 4.08E-03 |
| GO:1902905 | positive regulation of supramolecular fiber organization | BP | 4.25E-03 |
| GO:0034332 | adherens junction organization | BP | 4.45E-03 |
| GO:0031960 | response to corticosteroid | BP | 4.72E-03 |
| GO:0022602 | ovulation cycle process | BP | 4.73E-03 |
| GO:0031345 | negative regulation of cell projection organization | BP | 4.86E-03 |
| GO:0046883 | regulation of hormone secretion | BP | 5.04E-03 |
| GO:0071383 | cellular response to steroid hormone stimulus | BP | 5.07E-03 |
| GO:0023061 | signal release | BP | 5.17E-03 |
| GO:0048732 | gland development | BP | 5.46E-03 |
| GO:0008584 | male gonad development | BP | 5.46E-03 |
| GO:0045665 | negative regulation of neuron differentiation | BP | 5.79E-03 |
| GO:0046546 | development of primary male sexual characteristics | BP | 5.87E-03 |
| GO:0002576 | platelet degranulation | BP | 5.87E-03 |
| GO:0045682 | regulation of epidermis development | BP | 6.37E-03 |
| GO:0051271 | negative regulation of cellular component movement | BP | 6.37E-03 |
| GO:0046661 | male sex differentiation | BP | 6.40E-03 |
| GO:0050768 | negative regulation of neurogenesis | BP | 6.48E-03 |
| GO:0010769 | regulation of cell morphogenesis involved in differentiation | BP | 6.63E-03 |
| GO:0042339 | keratan sulfate metabolic process | BP | 7.26E-03 |
| GO:0006790 | sulfur compound metabolic process | BP | 8.16E-03 |
| GO:0031100 | animal organ regeneration | BP | 8.99E-03 |
| GO:0048871 | multicellular organismal homeostasis | BP | 8.99E-03 |
| GO:0008361 | regulation of cell size | BP | 8.99E-03 |
| GO:0002521 | leukocyte differentiation | BP | 8.99E-03 |
| GO:0032231 | regulation of actin filament bundle assembly | BP | 9.06E-03 |
| GO:0060996 | dendritic spine development | BP | 9.06E-03 |
| GO:0051656 | establishment of organelle localization | BP | 9.06E-03 |
| GO:0002446 | neutrophil mediated immunity | BP | 9.06E-03 |
| GO:0031346 | positive regulation of cell projection organization | BP | 9.06E-03 |
| GO:0051961 | negative regulation of nervous system development | BP | 9.06E-03 |
| GO:0045185 | maintenance of protein location | BP | 9.06E-03 |
| GO:0009150 | purine ribonucleotide metabolic process | BP | 9.14E-03 |
| GO:0007411 | axon guidance | BP | 9.14E-03 |
| GO:0030055 | cell-substrate junction | CC | 2.73E-05 |
| GO:0005925 | focal adhesion | CC | 2.73E-05 |
| GO:0005924 | cell-substrate adherens junction | CC | 2.73E-05 |
| GO:0005912 | adherens junction | CC | 2.76E-05 |
| GO:0042641 | actomyosin | CC | 3.00E-03 |
| GO:0043202 | lysosomal lumen | CC | 3.00E-03 |
| GO:0001726 | ruffle | CC | 4.10E-03 |
| GO:0001725 | stress fiber | CC | 5.36E-03 |
| GO:0097517 | contractile actin filament bundle | CC | 5.36E-03 |
| GO:0030667 | secretory granule membrane | CC | 6.91E-03 |

Table S2 The top 100 GO analysis of DEGs in HCC

| ID | Term | Category | P_value |
| --- | --- | --- | --- |
| GO:0016054 | organic acid catabolic process | BP | 2.83E-28 |
| GO:0046395 | carboxylic acid catabolic process | BP | 2.83E-28 |
| GO:0044282 | small molecule catabolic process | BP | 1.22E-25 |
| GO:0009063 | cellular amino acid catabolic process | BP | 1.23E-21 |
| GO:1901605 | alpha-amino acid metabolic process | BP | 1.14E-20 |
| GO:1901606 | alpha-amino acid catabolic process | BP | 1.40E-18 |
| GO:0016053 | organic acid biosynthetic process | BP | 1.68E-17 |
| GO:0046394 | carboxylic acid biosynthetic process | BP | 4.35E-17 |
| GO:0006520 | cellular amino acid metabolic process | BP | 4.66E-17 |
| GO:0006631 | fatty acid metabolic process | BP | 6.99E-15 |
| GO:0007059 | chromosome segregation | BP | 3.61E-13 |
| GO:0071466 | cellular response to xenobiotic stimulus | BP | 5.41E-13 |
| GO:0042737 | drug catabolic process | BP | 1.43E-12 |
| GO:0008202 | steroid metabolic process | BP | 1.55E-12 |
| GO:0009410 | response to xenobiotic stimulus | BP | 5.43E-12 |
| GO:0072329 | monocarboxylic acid catabolic process | BP | 8.00E-12 |
| GO:1901615 | organic hydroxy compound metabolic process | BP | 8.00E-12 |
| GO:0000070 | mitotic sister chromatid segregation | BP | 3.22E-11 |
| GO:0000819 | sister chromatid segregation | BP | 3.22E-11 |
| GO:0098813 | nuclear chromosome segregation | BP | 5.49E-11 |
| GO:0072330 | monocarboxylic acid biosynthetic process | BP | 7.82E-11 |
| GO:0009072 | aromatic amino acid family metabolic process | BP | 2.15E-10 |
| GO:0051983 | regulation of chromosome segregation | BP | 2.44E-10 |
| GO:0009074 | aromatic amino acid family catabolic process | BP | 2.53E-10 |
| GO:0140014 | mitotic nuclear division | BP | 2.71E-10 |
| GO:0006260 | DNA replication | BP | 3.08E-10 |
| GO:0006732 | coenzyme metabolic process | BP | 6.32E-10 |
| GO:0043648 | dicarboxylic acid metabolic process | BP | 1.06E-09 |
| GO:0048285 | organelle fission | BP | 1.06E-09 |
| GO:0010038 | response to metal ion | BP | 1.93E-09 |
| GO:0006066 | alcohol metabolic process | BP | 3.20E-09 |
| GO:0006805 | xenobiotic metabolic process | BP | 4.69E-09 |
| GO:0001101 | response to acid chemical | BP | 7.70E-09 |
| GO:0000280 | nuclear division | BP | 1.04E-08 |
| GO:0009991 | response to extracellular stimulus | BP | 1.67E-08 |
| GO:0044843 | cell cycle G1,S phase transition | BP | 4.69E-08 |
| GO:0006006 | glucose metabolic process | BP | 5.16E-08 |
| GO:1901568 | fatty acid derivative metabolic process | BP | 5.22E-08 |
| GO:0000075 | cell cycle checkpoint | BP | 5.22E-08 |
| GO:0008652 | cellular amino acid biosynthetic process | BP | 7.03E-08 |
| GO:0009066 | aspartate family amino acid metabolic process | BP | 7.81E-08 |
| GO:0000082 | G1,S transition of mitotic cell cycle | BP | 7.81E-08 |
| GO:0044242 | cellular lipid catabolic process | BP | 1.47E-07 |
| GO:0019318 | hexose metabolic process | BP | 2.79E-07 |
| GO:0009636 | response to toxic substance | BP | 4.44E-07 |
| GO:0009064 | glutamine family amino acid metabolic process | BP | 4.52E-07 |
| GO:0016042 | lipid catabolic process | BP | 4.76E-07 |
| GO:0007091 | metaphase,anaphase transition of mitotic cell cycle | BP | 5.14E-07 |
| GO:0010965 | regulation of mitotic sister chromatid separation | BP | 5.14E-07 |
| GO:0019373 | epoxygenase P450 pathway | BP | 5.14E-07 |
| GO:0051188 | cofactor biosynthetic process | BP | 5.38E-07 |
| GO:0001676 | long-chain fatty acid metabolic process | BP | 6.77E-07 |
| GO:0033045 | regulation of sister chromatid segregation | BP | 7.23E-07 |
| GO:0031099 | regeneration | BP | 8.76E-07 |
| GO:0044784 | metaphase,anaphase transition of cell cycle | BP | 9.96E-07 |
| GO:0051306 | mitotic sister chromatid separation | BP | 9.96E-07 |
| GO:0031667 | response to nutrient levels | BP | 1.08E-06 |
| GO:0006261 | DNA-dependent DNA replication | BP | 1.10E-06 |
| GO:0005996 | monosaccharide metabolic process | BP | 1.29E-06 |
| GO:1902652 | secondary alcohol metabolic process | BP | 1.80E-06 |
| GO:0016125 | sterol metabolic process | BP | 2.00E-06 |
| GO:0019395 | fatty acid oxidation | BP | 2.08E-06 |
| GO:0042180 | cellular ketone metabolic process | BP | 2.23E-06 |
| GO:0072001 | renal system development | BP | 2.34E-06 |
| GO:0009062 | fatty acid catabolic process | BP | 2.50E-06 |
| GO:1905818 | regulation of chromosome separation | BP | 2.50E-06 |
| GO:0008203 | cholesterol metabolic process | BP | 2.85E-06 |
| GO:0034440 | lipid oxidation | BP | 3.01E-06 |
| GO:0009108 | coenzyme biosynthetic process | BP | 3.40E-06 |
| GO:1902850 | microtubule cytoskeleton organization involved in mitosis | BP | 3.49E-06 |
| GO:0007051 | spindle organization | BP | 5.29E-06 |
| GO:0007093 | mitotic cell cycle checkpoint | BP | 5.29E-06 |
| GO:0030071 | regulation of mitotic metaphase,anaphase transition | BP | 5.97E-06 |
| GO:0006323 | DNA packaging | BP | 6.27E-06 |
| GO:0002526 | acute inflammatory response | BP | 8.19E-06 |
| GO:0007088 | regulation of mitotic nuclear division | BP | 8.25E-06 |
| GO:0006270 | DNA replication initiation | BP | 8.34E-06 |
| GO:0072376 | protein activation cascade | BP | 9.45E-06 |
| GO:0031100 | animal organ regeneration | BP | 1.00E-05 |
| GO:0033047 | regulation of mitotic sister chromatid segregation | BP | 1.03E-05 |
| GO:0000775 | chromosome, centromeric region | CC | 9.93E-12 |
| GO:0098687 | chromosomal region | CC | 9.93E-12 |
| GO:0000793 | condensed chromosome | CC | 4.27E-10 |
| GO:0031012 | extracellular matrix | CC | 2.51E-09 |
| GO:0000779 | condensed chromosome, centromeric region | CC | 2.51E-09 |
| GO:0000776 | kinetochore | CC | 3.21E-09 |
| GO:0000777 | condensed chromosome kinetochore | CC | 3.21E-09 |
| GO:0062023 | collagen-containing extracellular matrix | CC | 3.40E-09 |
| GO:0005819 | spindle | CC | 2.94E-08 |
| GO:0072686 | mitotic spindle | CC | 1.34E-07 |
| GO:0048037 | cofactor binding | MF | 5.57E-20 |
| GO:0050662 | coenzyme binding | MF | 8.26E-18 |
| GO:0004497 | monooxygenase activity | MF | 1.59E-08 |
| GO:0005506 | iron ion binding | MF | 1.59E-06 |
| GO:0016705 | oxidoreductase activity, acting on paired donors, with incorporation or reduction of molecular oxygen | MF | 4.62E-06 |
| GO:0020037 | heme binding | MF | 5.91E-06 |
| GO:0005539 | glycosaminoglycan binding | MF | 5.91E-06 |
| GO:0008483 | transaminase activity | MF | 1.33E-05 |
| GO:0050660 | flavin adenine dinucleotide binding | MF | 1.51E-05 |
| GO:0016614 | oxidoreductase activity, acting on CH-OH group of donors | MF | 1.73E-05 |

Table S3 The top 20 KEGG analysis of upregulated DEGs inT2DM

| ID | Term | P_value |
| --- | --- | --- |
| hsa04610 | Complement and coagulation cascades | 6.29E-06 |
| hsa04060 | Cytokine-cytokine receptor interaction | 4.83E-05 |
| hsa05200 | Pathways in cancer | 1.73E-04 |
| hsa04064 | NF-kappa B signaling pathway | 4.30E-04 |
| hsa00590 | Arachidonic acid metabolism | 7.36E-04 |
| hsa04080 | Neuroactive ligand-receptor interaction | 1.69E-03 |
| hsa04512 | ECM-receptor interaction | 6.08E-03 |
| hsa04151 | PI3K-Akt signaling pathway | 9.10E-03 |
| hsa04510 | Focal adhesion | 1.27E-02 |
| hsa00830 | Retinol metabolism | 1.86E-02 |
| hsa05323 | Rheumatoid arthritis | 1.88E-02 |
| hsa04062 | Chemokine signaling pathway | 2.04E-02 |
| hsa05144 | Malaria | 2.17E-02 |
| hsa05205 | Proteoglycans in cancer | 2.25E-02 |
| hsa04380 | Osteoclast differentiation | 2.38E-02 |
| hsa05340 | Primary immunodeficiency | 2.42E-02 |
| hsa04360 | Axon guidance | 4.12E-02 |
| hsa00230 | Purine metabolism | 4.18E-02 |
| hsa05410 | Hypertrophic cardiomyopathy (HCM) | 4.31E-02 |
| hsa05032 | Morphine addiction | 4.75E-02 |

Table S4 The top 20 KEGG analysis of downregulated DEGs inT2DM

| ID | Term | P_value |
| --- | --- | --- |
| hsa01100 | Metabolic pathways | 2.95E-07 |
| hsa00280 | Valine, leucine and isoleucine degradation | 3.88E-07 |
| hsa01130 | Biosynthesis of antibiotics | 5.49E-05 |
| hsa00071 | Fatty acid degradation | 8.13E-04 |
| hsa00620 | Pyruvate metabolism | 8.13E-04 |
| hsa00190 | Oxidative phosphorylation | 1.28E-03 |
| hsa04140 | Regulation of autophagy | 1.48E-03 |
| hsa04728 | Dopaminergic synapse | 1.74E-03 |
| hsa01212 | Fatty acid metabolism | 2.71E-03 |
| hsa04144 | Endocytosis | 2.95E-03 |
| hsa00010 | Glycolysis / Gluconeogenesis | 3.84E-03 |
| hsa04721 | Synaptic vesicle cycle | 4.28E-03 |
| hsa04142 | Lysosome | 5.47E-03 |
| hsa00650 | Butanoate metabolism | 7.09E-03 |
| hsa04922 | Glucagon signaling pathway | 8.82E-03 |
| hsa04911 | Insulin secretion | 9.56E-03 |
| hsa00520 | Amino sugar and nucleotide sugar metabolism | 1.04E-02 |
| hsa01200 | Carbon metabolism | 1.06E-02 |
| hsa00640 | Propanoate metabolism | 1.34E-02 |
| hsa00630 | Glyoxylate and dicarboxylate metabolism | 1.34E-02 |

Table S5 The top 16 KEGG analysis of upregulated DEGs in HCC

| ID | Term | P_value |
| --- | --- | --- |
| hsa04110 | Cell cycle | 2.59E-21 |
| hsa03030 | DNA replication | 6.41E-11 |
| hsa03460 | Fanconi anemia pathway | 1.15E-06 |
| hsa05222 | Small cell lung cancer | 2.53E-05 |
| hsa04114 | Oocyte meiosis | 3.48E-04 |
| hsa04115 | p53 signaling pathway | 3.87E-04 |
| hsa00240 | Pyrimidine metabolism | 2.11E-03 |
| hsa04512 | ECM-receptor interaction | 2.59E-03 |
| hsa05203 | Viral carcinogenesis | 3.94E-03 |
| hsa05322 | Systemic lupus erythematosus | 5.59E-03 |
| hsa03430 | Mismatch repair | 6.84E-03 |
| hsa05200 | Pathways in cancer | 1.35E-02 |
| hsa03410 | Base excision repair | 2.43E-02 |
| hsa04914 | Progesterone-mediated oocyte maturation | 2.75E-02 |
| hsa05034 | Alcoholism | 3.76E-02 |
| hsa05219 | Bladder cancer | 4.88E-02 |

Table S6 The top 30 KEGG analysis of downregulated DEGs in HCC

| ID | Term | P_value |
| --- | --- | --- |
| hsa01100 | Metabolic pathways | 3.89E-19 |
| hsa00380 | Tryptophan metabolism | 8.59E-11 |
| hsa04610 | Complement and coagulation cascades | 2.91E-10 |
| hsa00071 | Fatty acid degradation | 3.73E-10 |
| hsa00280 | Valine, leucine and isoleucine degradation | 4.59E-09 |
| hsa00250 | Alanine, aspartate and glutamate metabolism | 1.54E-08 |
| hsa01130 | Biosynthesis of antibiotics | 6.88E-08 |
| hsa00650 | Butanoate metabolism | 6.38E-07 |
| hsa00350 | Tyrosine metabolism | 1.74E-06 |
| hsa00410 | beta-Alanine metabolism | 2.21E-06 |
| hsa01200 | Carbon metabolism | 2.22E-06 |
| hsa05204 | Chemical carcinogenesis | 6.07E-06 |
| hsa00640 | Propanoate metabolism | 8.53E-06 |
| hsa00260 | Glycine, serine and threonine metabolism | 1.13E-05 |
| hsa05020 | Prion diseases | 4.41E-05 |
| hsa01212 | Fatty acid metabolism | 6.42E-05 |
| hsa00982 | Drug metabolism - cytochrome P450 | 1.31E-04 |
| hsa00220 | Arginine biosynthesis | 1.61E-04 |
| hsa00340 | Histidine metabolism | 3.30E-04 |
| hsa00630 | Glyoxylate and dicarboxylate metabolism | 4.07E-04 |
| hsa03320 | PPAR signaling pathway | 6.67E-04 |
| hsa04146 | Peroxisome | 7.07E-04 |
| hsa00983 | Drug metabolism - other enzymes | 8.14E-04 |
| hsa00620 | Pyruvate metabolism | 9.95E-04 |
| hsa00232 | Caffeine metabolism | 1.20E-03 |
| hsa00140 | Steroid hormone biosynthesis | 1.71E-03 |
| hsa00310 | Lysine degradation | 1.71E-03 |
| hsa00830 | Retinol metabolism | 1.71E-03 |
| hsa00561 | Glycerolipid metabolism | 1.86E-03 |
| hsa04060 | Cytokine-cytokine receptor interaction | 2.57E-03 |

Table S7 The top 10 GO analysis of each module in T2DM

| Module | ID | Term | Category | P_value |
| --- | --- | --- | --- | --- |
| black | GO:0007186 | G protein-coupled receptor signaling pathway | BP | 1.01E-05 |
| black | GO:0007165 | signal transduction | BP | 1.20E-05 |
| black | GO:0007154 | cell communication | BP | 4.71E-05 |
| black | GO:0023052 | signaling | BP | 5.20E-05 |
| black | GO:0002576 | platelet degranulation | BP | 9.19E-05 |
| black | GO:0040017 | positive regulation of locomotion | BP | 1.03E-04 |
| black | GO:0050896 | response to stimulus | BP | 1.65E-04 |
| black | GO:0001501 | skeletal system development | BP | 1.95E-04 |
| black | GO:0001503 | ossification | BP | 1.95E-04 |
| black | GO:0007596 | blood coagulation | BP | 2.06E-04 |
| blue | GO:0007005 | mitochondrion organization | BP | 6.42E-04 |
| blue | GO:0016246 | RNA interference | BP | 7.56E-03 |
| blue | GO:0030422 | production of siRNA involved in RNA interference | BP | 7.56E-03 |
| blue | GO:0043653 | mitochondrial fragmentation involved in apoptotic process | BP | 7.56E-03 |
| blue | GO:0045116 | protein neddylation | BP | 7.56E-03 |
| blue | GO:0043170 | macromolecule metabolic process | BP | 8.86E-03 |
| blue | GO:0016070 | RNA metabolic process | BP | 9.66E-03 |
| blue | GO:0031123 | RNA 3'-end processing | BP | 1.07E-02 |
| blue | GO:0042149 | cellular response to glucose starvation | BP | 1.07E-02 |
| blue | GO:0000045 | autophagosome assembly | BP | 1.12E-02 |
| brown | GO:2000669 | negative regulation of dendritic cell apoptotic process | BP | 1.32E-04 |
| brown | GO:0000070 | mitotic sister chromatid segregation | BP | 3.69E-04 |
| brown | GO:0001768 | establishment of T cell polarity | BP | 3.90E-04 |
| brown | GO:0002408 | myeloid dendritic cell chemotaxis | BP | 3.90E-04 |
| brown | GO:0033197 | response to vitamin E | BP | 3.90E-04 |
| brown | GO:0097026 | dendritic cell dendrite assembly | BP | 3.90E-04 |
| brown | GO:2000547 | regulation of dendritic cell dendrite assembly | BP | 3.90E-04 |
| brown | GO:0030010 | establishment of cell polarity | BP | 6.03E-04 |
| brown | GO:0046395 | carboxylic acid catabolic process | BP | 6.26E-04 |
| brown | GO:0009060 | aerobic respiration | BP | 8.21E-04 |
| green | GO:0006119 | oxidative phosphorylation | BP | 2.97E-11 |
| green | GO:0046034 | ATP metabolic process | BP | 1.42E-08 |
| green | GO:0042775 | mitochondrial ATP synthesis coupled electron transport | BP | 2.45E-07 |
| green | GO:0006163 | purine nucleotide metabolic process | BP | 4.11E-07 |
| green | GO:0009117 | nucleotide metabolic process | BP | 4.80E-07 |
| green | GO:0019693 | ribose phosphate metabolic process | BP | 5.16E-07 |
| green | GO:0022904 | respiratory electron transport chain | BP | 9.54E-07 |
| green | GO:0006091 | generation of precursor metabolites and energy | BP | 1.30E-06 |
| green | GO:0022900 | electron transport chain | BP | 5.49E-06 |
| green | GO:0045333 | cellular respiration | BP | 7.68E-06 |
| grey | GO:0002684 | positive regulation of immune system process | BP | 4.14E-04 |
| grey | GO:0007155 | cell adhesion | BP | 5.76E-04 |
| grey | GO:0006952 | defense response | BP | 1.05E-03 |
| grey | GO:0098609 | cell-cell adhesion | BP | 1.12E-03 |
| grey | GO:0000731 | DNA synthesis involved in DNA repair | BP | 1.15E-03 |
| grey | GO:0006301 | postreplication repair | BP | 1.15E-03 |
| grey | GO:0019985 | translesion synthesis | BP | 1.15E-03 |
| grey | GO:0050863 | regulation of T cell activation | BP | 1.32E-03 |
| grey | GO:0045785 | positive regulation of cell adhesion | BP | 1.42E-03 |
| grey | GO:0030155 | regulation of cell adhesion | BP | 1.54E-03 |
| pink | GO:0048870 | cell motility | BP | 5.20E-07 |
| pink | GO:0030198 | extracellular matrix organization | BP | 7.73E-07 |
| pink | GO:0016477 | cell migration | BP | 1.08E-06 |
| pink | GO:0040011 | locomotion | BP | 1.17E-06 |
| pink | GO:0043062 | extracellular structure organization | BP | 4.83E-06 |
| pink | GO:0048660 | regulation of smooth muscle cell proliferation | BP | 1.15E-05 |
| pink | GO:0050727 | regulation of inflammatory response | BP | 1.41E-05 |
| pink | GO:0048659 | smooth muscle cell proliferation | BP | 1.64E-05 |
| pink | GO:0008283 | cell proliferation | BP | 1.89E-05 |
| pink | GO:0007166 | cell surface receptor signaling pathway | BP | 1.92E-05 |
| red | GO:0001676 | long-chain fatty acid metabolic process | BP | 2.40E-05 |
| red | GO:0036101 | leukotriene B4 catabolic process | BP | 8.80E-05 |
| red | GO:0042758 | long-chain fatty acid catabolic process | BP | 8.80E-05 |
| red | GO:0006952 | defense response | BP | 9.44E-05 |
| red | GO:0006690 | icosanoid metabolic process | BP | 1.55E-04 |
| red | GO:0043616 | keratinocyte proliferation | BP | 2.31E-04 |
| red | GO:0006766 | vitamin metabolic process | BP | 3.37E-04 |
| red | GO:0042182 | ketone catabolic process | BP | 3.41E-04 |
| red | GO:0032787 | monocarboxylic acid metabolic process | BP | 3.60E-04 |
| red | GO:0019369 | arachidonic acid metabolic process | BP | 4.01E-04 |
| turquoise | GO:0048858 | cell projection morphogenesis | BP | 2.01E-04 |
| turquoise | GO:0007409 | cell development | BP | 6.22E-04 |
| turquoise | GO:0048812 | neuron projection morphogenesis | BP | 2.07E-04 |
| turquoise | GO:0007399 | nervous system development | BP | 2.26E-04 |
| turquoise | GO:0032990 | cell part morphogenesis | BP | 3.98E-04 |
| turquoise | GO:0048667 | cell morphogenesis involved in differentiation | BP | 1.00E-03 |
| turquoise | GO:0048699 | generation of neurons | BP | 7.01E-04 |
| turquoise | GO:0048666 | neuron development | BP | 7.39E-04 |
| turquoise | GO:0030182 | neuron differentiation | BP | 8.70E-04 |
| turquoise | GO:0031175 | regulation of cellular process | BP | 6.08E-04 |
| yellow | GO:0030522 | intracellular receptor signaling pathway | BP | 3.07E-05 |
| yellow | GO:0032922 | circadian regulation of gene expression | BP | 3.88E-05 |
| yellow | GO:0007623 | circadian rhythm | BP | 1.49E-04 |
| yellow | GO:0016070 | RNA metabolic process | BP | 4.64E-04 |
| yellow | GO:0010629 | negative regulation of gene expression | BP | 5.35E-04 |
| yellow | GO:0031324 | negative regulation of cellular metabolic process | BP | 6.55E-04 |
| yellow | GO:0060070 | canonical Wnt signaling pathway | BP | 8.96E-04 |
| yellow | GO:0016567 | protein ubiquitination | BP | 1.02E-03 |
| yellow | GO:0045892 | negative regulation of transcription, DNA-templated | BP | 1.15E-03 |
| yellow | GO:0042752 | regulation of circadian rhythm | BP | 1.35E-03 |

Table S8 The top 10 GO analysis of each module in HCC

| Module | ID | Term | Category | P_value |
| --- | --- | --- | --- | --- |
| blue | GO:0023052 | signaling | BP | 4.09E-07 |
| blue | GO:0007154 | cell communication | BP | 8.31E-07 |
| blue | GO:0007165 | signal transduction | BP | 1.65E-06 |
| blue | GO:0048731 | system development | BP | 2.68E-06 |
| blue | GO:0001818 | negative regulation of cytokine production | BP | 6.61E-06 |
| blue | GO:0007275 | multicellular organism development | BP | 1.49E-05 |
| blue | GO:0001944 | vasculature development | BP | 2.35E-05 |
| blue | GO:0071310 | cellular response to organic substance | BP | 2.69E-05 |
| blue | GO:0001568 | blood vessel development | BP | 2.75E-05 |
| blue | GO:0032502 | developmental process | BP | 2.99E-05 |
| brown | GO:0006082 | organic acid metabolic process | BP | 4.28E-09 |
| brown | GO:0044281 | small molecule metabolic process | BP | 1.07E-07 |
| brown | GO:0006790 | sulfur compound metabolic process | BP | 2.30E-07 |
| brown | GO:0019752 | carboxylic acid metabolic process | BP | 4.48E-07 |
| brown | GO:0055114 | oxidation-reduction process | BP | 1.64E-06 |
| brown | GO:0046395 | carboxylic acid catabolic process | BP | 5.13E-06 |
| brown | GO:0017144 | drug metabolic process | BP | 8.20E-06 |
| brown | GO:0042737 | drug catabolic process | BP | 4.94E-05 |
| brown | GO:0009056 | catabolic process | BP | 1.51E-04 |
| brown | GO:0051186 | cofactor metabolic process | BP | 2.44E-04 |
| green | GO:0006955 | immune response | BP | 1.63E-13 |
| green | GO:0002376 | immune system process | BP | 4.51E-10 |
| green | GO:0050776 | regulation of immune response | BP | 3.34E-09 |
| green | GO:0002274 | myeloid leukocyte activation | BP | 4.92E-09 |
| green | GO:0045321 | leukocyte activation | BP | 6.05E-09 |
| green | GO:0007259 | JAK-STAT cascade | BP | 1.61E-08 |
| green | GO:0097696 | STAT cascade | BP | 1.61E-08 |
| green | GO:0001775 | cell activation | BP | 2.00E-08 |
| green | GO:0045088 | regulation of innate immune response | BP | 2.29E-08 |
| green | GO:0002682 | regulation of immune system process | BP | 2.49E-08 |
| grey | GO:0072086 | specification of loop of Henle identity | BP | 1.29E-03 |
| grey | GO:0009954 | proximal/distal pattern formation | BP | 2.57E-03 |
| grey | GO:0072070 | loop of Henle development | BP | 2.57E-03 |
| grey | GO:0072079 | nephron tubule formation | BP | 2.57E-03 |
| grey | GO:0007379 | segment specification | BP | 3.86E-03 |
| grey | GO:0072028 | nephron morphogenesis | BP | 1.16E-02 |
| grey | GO:0097009 | energy homeostasis | BP | 1.16E-02 |
| grey | GO:0061333 | renal tubule morphogenesis | BP | 1.28E-02 |
| grey | GO:0060993 | kidney morphogenesis | BP | 1.41E-02 |
| grey | GO:0001838 | embryonic epithelial tube formation | BP | 1.54E-02 |
| red | GO:0030198 | extracellular matrix organization | BP | 2.13E-13 |
| red | GO:0043062 | extracellular structure organization | BP | 3.87E-12 |
| red | GO:0007155 | cell adhesion | BP | 2.50E-08 |
| red | GO:0007166 | cell surface receptor signaling pathway | BP | 2.71E-07 |
| red | GO:0070848 | response to growth factor | BP | 4.44E-07 |
| red | GO:0009653 | anatomical structure morphogenesis | BP | 8.11E-07 |
| red | GO:0097435 | supramolecular fiber organization | BP | 8.55E-07 |
| red | GO:0009887 | animal organ morphogenesis | BP | 1.72E-06 |
| red | GO:0050896 | response to stimulus | BP | 6.02E-06 |
| red | GO:0060326 | cell chemotaxis | BP | 9.56E-06 |
| turquoise | GO:0007049 | cell cycle | BP | 9.93E-27 |
| turquoise | GO:0000278 | mitotic cell cycle | BP | 1.17E-25 |
| turquoise | GO:0051276 | chromosome organization | BP | 1.40E-23 |
| turquoise | GO:0051301 | cell division | BP | 5.57E-20 |
| turquoise | GO:0006259 | DNA metabolic process | BP | 3.02E-19 |
| turquoise | GO:0044772 | mitotic cell cycle phase transition | BP | 1.40E-17 |
| turquoise | GO:0044770 | cell cycle phase transition | BP | 5.56E-17 |
| turquoise | GO:0007059 | chromosome segregation | BP | 8.77E-16 |
| turquoise | GO:0140014 | mitotic nuclear division | BP | 8.00E-14 |
| turquoise | GO:0006260 | DNA replication | BP | 1.01E-13 |
| yellow | GO:0032787 | monocarboxylic acid metabolic process | BP | 1.25E-07 |
| yellow | GO:0019752 | carboxylic acid metabolic process | BP | 1.38E-07 |
| yellow | GO:0006082 | organic acid metabolic process | BP | 1.67E-07 |
| yellow | GO:0044281 | small molecule metabolic process | BP | 3.56E-07 |
| yellow | GO:0019373 | epoxygenase P450 pathway | BP | 9.65E-06 |
| yellow | GO:0019627 | urea metabolic process | BP | 2.76E-05 |
| yellow | GO:0036101 | leukotriene B4 catabolic process | BP | 2.76E-05 |
| yellow | GO:0042758 | long-chain fatty acid catabolic process | BP | 2.76E-05 |
| yellow | GO:0017144 | drug metabolic process | BP | 4.12E-05 |
| yellow | GO:0044255 | cellular lipid metabolic process | BP | 7.48E-05 |

Table S9 The top 100 functional analysis of 37 genes via Metascape

| ID | Term | Category | P_value |
| --- | --- | --- | --- |
| GO:0046395 | carboxylic acid catabolic process | GO BP | 1.00E-11 |
| GO:0016054 | organic acid catabolic process | GO BP | 1.00E-11 |
| GO:0006631 | fatty acid metabolic process | GO BP | 6.31E-10 |
| GO:0072329 | monocarboxylic acid catabolic process | GO BP | 2.00E-09 |
| GO:0044282 | small molecule catabolic process | GO BP | 2.51E-09 |
| GO:0032787 | monocarboxylic acid metabolic process | GO BP | 6.31E-09 |
| hsa00071 | Fatty acid degradation | KEGG Pathway | 1.00E-06 |
| GO:0009062 | fatty acid catabolic process | GO BP | 1.00E-06 |
| hsa01212 | Fatty acid metabolism | KEGG Pathway | 1.26E-06 |
| hsa00280 | Valine, leucine and isoleucine degradation | KEGG Pathway | 1.26E-06 |
| GO:0044242 | cellular lipid catabolic process | GO BP | 1.58E-06 |
| GO:0006520 | cellular amino acid metabolic process | GO BP | 2.00E-06 |
| GO:0009063 | cellular amino acid catabolic process | GO BP | 2.00E-06 |
| GO:0001889 | liver development | GO BP | 3.98E-06 |
| GO:0061008 | hepaticobiliary system development | GO BP | 3.98E-06 |
| GO:0006635 | fatty acid beta-oxidation | GO BP | 6.31E-06 |
| GO:0043434 | response to peptide hormone | GO BP | 7.94E-06 |
| hsa00650 | Butanoate metabolism | KEGG Pathway | 1.26E-05 |
| GO:0016042 | lipid catabolic process | GO BP | 2.00E-05 |
| GO:0019395 | fatty acid oxidation | GO BP | 2.51E-05 |
| GO:1901652 | response to peptide | GO BP | 2.51E-05 |
| GO:0034440 | lipid oxidation | GO BP | 3.16E-05 |
| GO:0033762 | response to glucagon | GO BP | 3.16E-05 |
| hsa00620 | Pyruvate metabolism | KEGG Pathway | 3.98E-05 |
| GO:0030258 | lipid modification | GO BP | 5.01E-05 |
| GO:0006732 | coenzyme metabolic process | GO BP | 6.31E-05 |
| M106 | PID HNF3B PATHWAY | Canonical Pathways | 6.31E-05 |
| GO:0019216 | regulation of lipid metabolic process | GO BP | 6.31E-05 |
| GO:0009108 | coenzyme biosynthetic process | GO BP | 7.94E-05 |
| GO:0005975 | carbohydrate metabolic process | GO BP | 7.94E-05 |
| GO:0048732 | gland development | GO BP | 1.00E-04 |
| GO:1990845 | adaptive thermogenesis | GO BP | 1.26E-04 |
| GO:0051188 | cofactor biosynthetic process | GO BP | 2.00E-04 |
| GO:0001659 | temperature homeostasis | GO BP | 2.00E-04 |
| R-HSA-556833 | Metabolism of lipids | Reactome Gene Sets | 2.00E-04 |
| hsa04920 | Adipocytokine signaling pathway | KEGG Pathway | 2.00E-04 |
| GO:0050796 | regulation of insulin secretion | GO BP | 2.00E-04 |
| GO:0006091 | generation of precursor metabolites and energy | GO BP | 2.00E-04 |
| GO:0046165 | alcohol biosynthetic process | GO BP | 2.51E-04 |
| GO:0005977 | glycogen metabolic process | GO BP | 2.51E-04 |
| GO:0044042 | glucan metabolic process | GO BP | 2.51E-04 |
| GO:0006073 | cellular glucan metabolic process | GO BP | 2.51E-04 |
| GO:0046173 | polyol biosynthetic process | GO BP | 2.51E-04 |
| GO:1901615 | organic hydroxy compound metabolic process | GO BP | 3.16E-04 |
| GO:0006066 | alcohol metabolic process | GO BP | 3.98E-04 |
| GO:0030073 | insulin secretion | GO BP | 3.98E-04 |
| GO:0006112 | energy reserve metabolic process | GO BP | 3.98E-04 |
| GO:0090276 | regulation of peptide hormone secretion | GO BP | 3.98E-04 |
| GO:0051186 | cofactor metabolic process | GO BP | 5.01E-04 |
| GO:1901605 | alpha-amino acid metabolic process | GO BP | 5.01E-04 |
| GO:0120162 | positive regulation of cold-induced thermogenesis | GO BP | 6.31E-04 |
| GO:0044264 | cellular polysaccharide metabolic process | GO BP | 6.31E-04 |
| GO:0006641 | triglyceride metabolic process | GO BP | 7.94E-04 |
| GO:1901606 | alpha-amino acid catabolic process | GO BP | 7.94E-04 |
| hsa04931 | Insulin resistance | KEGG Pathway | 7.94E-04 |
| GO:0030072 | peptide hormone secretion | GO BP | 7.94E-04 |
| GO:0005976 | polysaccharide metabolic process | GO BP | 7.94E-04 |
| GO:1904659 | glucose transmembrane transport | GO BP | 7.94E-04 |
| GO:0008645 | hexose transmembrane transport | GO BP | 1.00E-03 |
| GO:0015749 | monosaccharide transmembrane transport | GO BP | 1.00E-03 |
| GO:0034219 | carbohydrate transmembrane transport | GO BP | 1.00E-03 |
| GO:0046883 | regulation of hormone secretion | GO BP | 1.00E-03 |
| GO:1901617 | organic hydroxy compound biosynthetic process | GO BP | 1.26E-03 |
| GO:0008610 | lipid biosynthetic process | GO BP | 1.26E-03 |
| GO:0032868 | response to insulin | GO BP | 1.26E-03 |
| GO:0015980 | energy derivation by oxidation of organic compounds | GO BP | 1.26E-03 |
| GO:0019751 | polyol metabolic process | GO BP | 1.26E-03 |
| GO:0048871 | multicellular organismal homeostasis | GO BP | 1.26E-03 |
| GO:0006638 | neutral lipid metabolic process | GO BP | 1.26E-03 |
| GO:0006639 | acylglycerol metabolic process | GO BP | 1.26E-03 |
| GO:0050708 | regulation of protein secretion | GO BP | 1.26E-03 |
| GO:0009410 | response to xenobiotic stimulus | GO BP | 1.26E-03 |
| GO:0044262 | cellular carbohydrate metabolic process | GO BP | 1.26E-03 |
| hsa04068 | FoxO signaling pathway | KEGG Pathway | 1.58E-03 |
| GO:0031667 | response to nutrient levels | GO BP | 1.58E-03 |
| GO:0005996 | monosaccharide metabolic process | GO BP | 1.58E-03 |
| R-HSA-71387 | Metabolism of carbohydrates | Reactome Gene Sets | 1.58E-03 |
| hsa04910 | Insulin signaling pathway | KEGG Pathway | 1.58E-03 |
| R-HSA-446203 | Asparagine N-linked glycosylation | Reactome Gene Sets | 1.58E-03 |
| GO:0002791 | regulation of peptide secretion | GO BP | 1.58E-03 |
| GO:1901137 | carbohydrate derivative biosynthetic process | GO BP | 1.58E-03 |
| GO:0106106 | cold-induced thermogenesis | GO BP | 2.00E-03 |
| GO:0120161 | regulation of cold-induced thermogenesis | GO BP | 2.00E-03 |
| GO:0042737 | drug catabolic process | GO BP | 2.00E-03 |
| GO:0046879 | hormone secretion | GO BP | 2.00E-03 |
| GO:0009991 | response to extracellular stimulus | GO BP | 2.00E-03 |
| GO:0008643 | carbohydrate transport | GO BP | 2.00E-03 |
| GO:0009914 | hormone transport | GO BP | 2.00E-03 |
| GO:0071375 | cellular response to peptide hormone stimulus | GO BP | 2.00E-03 |
| GO:0030509 | BMP signaling pathway | GO BP | 2.51E-03 |
| GO:0034762 | regulation of transmembrane transport | GO BP | 2.51E-03 |
| GO:0001101 | response to acid chemical | GO BP | 2.51E-03 |
| GO:0015718 | monocarboxylic acid transport | GO BP | 3.16E-03 |
| GO:0071772 | response to BMP | GO BP | 3.16E-03 |
| GO:0071773 | cellular response to BMP stimulus | GO BP | 3.16E-03 |
| GO:0001666 | response to hypoxia | GO BP | 3.16E-03 |
| GO:1901568 | fatty acid derivative metabolic process | GO BP | 3.16E-03 |
| R-HSA-8978868 | Fatty acid metabolism | Reactome Gene Sets | 3.16E-03 |
| GO:0036293 | response to decreased oxygen levels | GO BP | 3.16E-03 |
| GO:0006790 | sulfur compound metabolic process | GO BP | 3.16E-03 |
